# A rare case of abnormal uterine bleeding caused by cavernous hemangioma: a case report

**DOI:** 10.1186/1752-1947-4-136

**Published:** 2010-05-17

**Authors:** Mridula A Benjamin, Hjh Roselina Yaakub, PU Telesinghe, Gazala Kafeel

**Affiliations:** 1 Department of Obstetrics and Gynecology, RIPAS Hospital, Bandar Seri Begawan, Brunei; 2 Department of Pathology, RIPAS Hospital, Bandar Seri Begawan, Brunei

## Abstract

**Introduction:**

Cavernous hemangiomas of the uterus are extremely rare, benign lesions. A survey of the current literature identified fewer than 50 cases of hemangioma of the uterus.

**Case presentation:**

We report a case of cavernous hemangioma of the uterus in a 27-year-old Malay, para 1 woman who presented at our hospital with torrential vaginal bleeding having been transferred by land ambulance from a district hospital 30 minutes away. 11 weeks previously she had an urgent cesarean section at our hospital. She had to undergo a hysterectomy to control her bleeding after other measures were unsuccessful. A histopathological report confirmed a diffuse ramifying hemangioma of the cervix and uterus with left hematosalpinx.

**Conclusion:**

Most ramifying hemangioma lesions are asymptomatic and are found incidentally, but sometimes they may cause abnormal vaginal bleeding and hence should be included in the differential diagnosis of patients with vaginal bleeding. Hysterectomy is the primary mode of treatment in most symptomatic cases.

## Introduction

Cavernous hemangiomas of the uterus are extremely rare, benign lesions. A survey of the literature identified fewer than 50 cases of hemangioma of the uterus. Although they can be found at all levels of the uterine wall, including the serosa, myometrium and endometrium, most cases usually involve the myometrium diffusely. These lesions are associated with numerous obstetric and gynecological complications, ranging from intermenstrual spotting, menometrorrhagia and infertility to maternal and fetal demise from pronounced bleeding of the gravid uterus [1-4].

We present a case of a para 1 woman with a ramifying hemangioma of the uterus who presented with torrential bleeding per vaginam eleven weeks after Cesarean section.

## Case presentation

A 27-year-old Malay, para 1 woman had an urgent lower segment Cesarean section (LSCS) after secondary arrest of cervical dilation in April 2008 at the RIPAS Hospital. During the LSCS, extension of left side of incision injured the uterine artery, which was repaired and hemostasis was secured. The post-operative period was uneventful and she was discharged after four days. On discharge our patient was given a Depo-Provera (depot medroxyprogesterone acetate) injection for contraception. She was re-admitted 10 days later with a secondary postpartum hemorrhage, with a blood loss of around 50mL. She recovered with antibiotics, cefuroxime and Flagyl (metronidazole), and was discharged four days later. She continued to have minimal bleeding per vaginam periodically since the delivery, which was attributed to the Depo-Provera (depot medroxyprogesterone acetate). She did not have any significant previous medical or family history of a bleeding disorder.

She presented again at a regional hospital 11 weeks after the Cesarean section following attendance at a martial arts competition in a nearby district with heavy bleeding per vaginam and was transferred by land ambulance to the RIPAS Hospital. She was pale with cold clammy skin and her blood pressure was 80/50mmHg. Her abdomen was soft and non-tender and no mass was palpable. Vaginal examination revealed a normal size uterus, her cervical os was closed and bleeding was moderate by then. Her bleeding was controlled using oxytocics and supportive management. A repeat episode of bleeding one hour later resulted in shock and active resuscitation was carried out.

Repeat per speculum examination showed moderate bleeding coming through the cervical os with around 150mL of clots. No vaginal tear was seen. Ultrasonography showed an empty uterus with a small hypoechoic area in the pouch of Douglas which was most likely to have been clots. No obvious adnexal mass was seen.

A urine pregnancy test was negative. Her blood results showed the following: hemoglobin 108gm/L, platelet 140 × 10^9^/L, beta-human chorionic gonadotropin (β-HCG) <1.2 IU/L, activated partial thromboplastin time (aPTT) 44.4s/32s, prothrombin time (PT) 19.8s/12s, international normalized ratio (INR) 2.0.

As the cause of bleeding could not be determined from any coagulation disorder or observed from a scar site, further investigations, such as a computed tomography (CT) scan, were considered.

One hour later our patient started bleeding torrentially per vaginam again. Repeat speculum examination showed a possible active bleeding point from her cervix. She was immediately taken from the Accident and Emergency unit to an operating room. Under anaesthesia, heavy bleeding was seen through the cervical os on a speculum examination but the exact location could not be localized and so a laparotomy was decided on. Fifty milliliters of old blood was seen in the peritoneal cavity, with a left hematosalpinx. There was no bleeding at the LSCS scar site, though the left uterine angle was slightly necrotic. Her uterus, right tube and both ovaries were normal. A left salpingectomy was performed. Left internal iliac ligation was initially carried out after tracing the ureter. However, a right internal iliac artery ligation could not be carried out as the ureter was difficult to trace, therefore a right uterine artery ligation was performed instead. Our patient continued to bleed torrentially per vaginam and the decision to perform a hysterectomy was taken. After the hysterectomy, the bleeding was controlled and her abdomen was closed, with one pelvic and two paracolic drains in place. From her arrival to the end of surgery the total estimated blood loss was between three and four liters. She received 10 units of blood and six units of fresh frozen plasma.

Post-operatively she was kept on ventilatory support for two days in intensive care unit. She recovered slowly and after four days all the drains were removed and our patient returned to a full diet. She was discharged on day six post-hysterectomy. Patient was counselled after six weeks during post operative review by gynaecologist. She was explained the histopathological report and her unusual series of vaginal bleeding following her Cesarean section. At her review, one year later, she was psychologically well and was able to look after her healthy one-year-old child.

**Figure 1:**
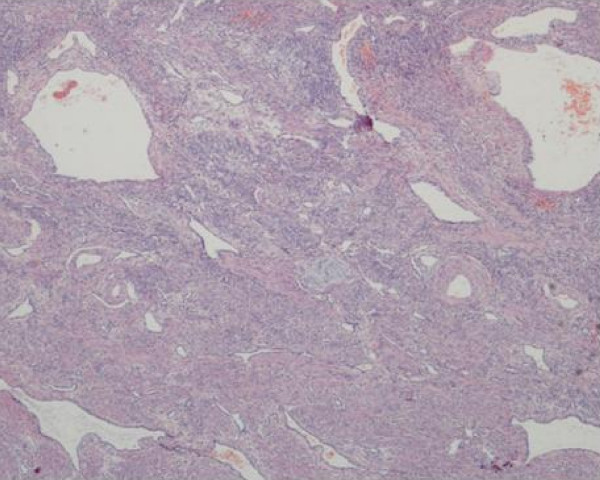
**Histopathology**. Endothelial lined vascular spaces were seen ramifying between the uterine musculature.

**Figure 2:**
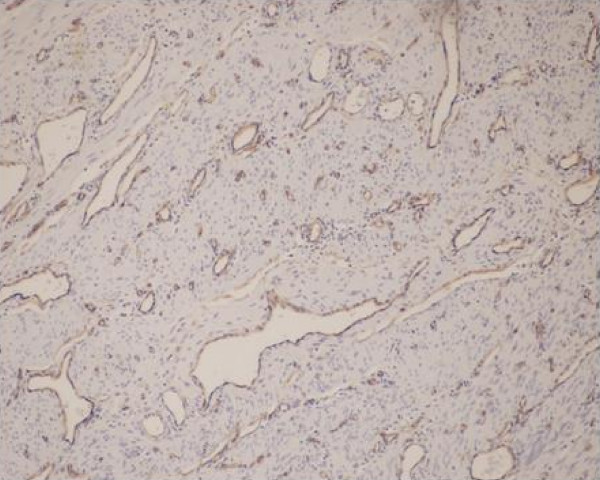
**CCD34 stain**. Brown endothelial cells confirming vascular spaces.

Histopathology examination showed a diffuse ramifying hemangioma of the cervix and uterus with left hematosalpinx. Endothelial lined vascular spaces were seen ramifying between the uterine musculature (Figure 1). CCD34 stained the endothelial cells brown (Figure 2). These were the diagnostic features of ramifying haemangioma which explained her torrential bleeding.

## Discussion

A differential diagnosis of diffuse ramifying hemangioma of the cervix and uterus was not determined during the initial resuscitation and diagnosis of our patient. This led to a delay in treatment and significant loss of blood. Earlier suspicion of this condition could have led to interventional measures during earlier clinic visits which could have resulted in the retention of her uterus and reduced morbidity. Hence the possibility of ramifying hemangiomas should be considered in the differential diagnosis of abnormal uterine bleeding where other causes have been ruled out.

All the cases of endometrial hemangiomas described in the literature to date have shown progressive symptoms of uterine bleeding which do not respond to conservative therapy [1-4]. Most of these lesions are asymptomatic and are found incidentally, but sometimes they may cause abnormal vaginal bleeding and hence should be included in the differential diagnosis of patients with vaginal bleeding.

With this condition, investigations such as vaginal examination, endometrial curettage, ultrasound, and hysterogram are non-informative and inconclusive. In a few cases the uterus has been reported to be pulsatile [5]. If there is any clinical suspicion in cases not responding to conservative treatment, a pelvic angiogram and CT may confirm the presence of a lesion. The treatment of uterine vascular anomalies that occur during pregnancy includes conservative measures such as close follow-up during the second and third trimesters, with close observation during delivery. Most patients have had successful vaginal and Cesarean deliveries despite the presence of extensive myometrial hemangiomas. The appropriate treatment for endometrial hemangiomas remains unclear. The few cases in the literature describe conservative treatments, such as carbon dioxide laser excision, knife excision, cryotherapy, radiotherapy, electrocauterization, and uterine artery embolization, having been tried. In cases not responding to conservative treatments, hysterectomy is to be considered. Non-surgical modalities such as radiotherapy would probably cure the lesions but in the process would destroy ovarian function [6].

## Conclusion

Most ramifying hemangioma lesions are asymptomatic and are found incidentally, but sometimes they may cause abnormal vaginal bleeding and hence should be included in the differential diagnosis of patients with vaginal bleeding. Hysterectomy is the primary mode of therapy in most symptomatic cases.

## Competing interests

The authors declare that they have no competing interests.

## Authors' contributions

MAB was the initial attending gynaecologist involved in the resuscitation, examination and surgery of our patient. HRY was the consultant obstetrician gynaecologist on call, and was involved in the surgery of the patient. GK was the pathologist involved in reporting the histopathology examination. PUT was the head of the Department of Pathology involved in confirming the histopathology report. All authors read and approved the final manuscript.

## Consent

Written informed consent was obtained from the patient for publication of this case report and any accompanying images. A copy of the written consent is available for review by the Editor-in-Chief of this journal.
